# Dizziness in postural tachycardia syndrome and its link to vestibular migraine

**DOI:** 10.3389/fneur.2025.1583348

**Published:** 2025-05-30

**Authors:** Ruby Sekhon, Nicholas Gall, Carolyn Ainsworth, Louisa Murdin

**Affiliations:** ^1^Guy's and St Thomas' NHS Foundation Trust, London, United Kingdom; ^2^Kings College Hospital NHS Foundation Trust, London, United Kingdom; ^3^Ear Institute, University College London, London, United Kingdom

**Keywords:** postural tachycardia syndrome, migraine, vertigo, clinical observation, dizziness

## Abstract

**Background:**

Postural tachycardia syndrome (PoTS) is a chronic condition characterized by an increased heart rate upon standing and is recognized to be associated with dizziness and migraines. This study aimed to investigate the multifaceted nature of dizziness in PoTS from a vestibular perspective, including the relationship with vestibular migraine.

**Methods:**

A retrospective review of 80 patients with confirmed PoTS, attending a tertiary neuro-otology clinic, was conducted to evaluate standardized detailed assessments {validated symptom questionnaires, history/physical examination, neuro-otological diagnostics [including video head impulse test (VHIT), videonystagmography (VNG), and caloric testing], and management}. The PoTS cohort was also compared with an age- and sex-matched control group of dizzy patients.

**Results:**

A total of 80 patients were included (mean age: 35.3 years ± 12.1; 93% women). Approximately 84% had migraine, and 30% had vestibular migraine. Clinical examination of static and dynamic balance was most frequently abnormal. VHIT was abnormal in 2%, and VNG canal paresis and directional preponderance were abnormal in 19 and 22%, respectively. In total, 49% of cases were offered migraine management advice, and 58% were offered vestibular rehabilitation. The PoTS group showed higher rates of vestibular symptoms, including vertigo, unsteadiness, and positional vertigo, and high Disability Rating Scale (DRS) scores.

**Conclusion:**

Our findings reflect the challenges of distinguishing dizziness phenotypes in PoTS, particularly vestibular migraine. This study illustrates the importance of judicious clinical enquiry to investigate non-orthostatic dizziness mechanisms, such as vestibular migraine, for which evidence-based management exists, and recommends further research to elucidate the multifaceted nature of PoTS-associated dizziness.

## Introduction

Postural tachycardia syndrome (PoTS, also known as postural orthostatic tachycardia syndrome [POTS]) is a chronic heterogeneous condition defined clinically by a minimum of two consecutive recordings demonstrating a sustained increase in heart rate of more than 30 beats per minute over 10 minutes of standing in the absence of orthostatic hypotension (1). The cardinal symptom of PoTS is dizziness, especially of orthostatic type. Haemodynamic orthostatic dizziness has been discussed in detail in a Barany Society Consensus document (2).

Widely recognized for its female preponderance, typically affecting those between 12 and 50 years of age, the prevalence of PoTS is not truly appreciated due to underdiagnosis (3, 4). Published estimates from the United States report a prevalence between 0.1 and 1% (4). Migraine, a common comorbidity in PoTS, is also well known as a cause of dizziness in neurology and neuro-otology clinics, primarily in the form of vestibular migraine (5). Association with Ehlers–Danlos Syndrome (EDS), hypermobility, fibromyalgia, depression, and anxiety further illustrate the complexity of the PoTS spectrum (6).

Currently, there is a paucity of evidence investigating the nature of dizziness in PoTS beyond orthostatic intolerance. This study, therefore, intended to examine dizziness in PoTS from a neuro-otological perspective to identify the prevalence of common dizziness presentations and conditions, including vestibular migraine (VM).

## Methods

### Design

A retrospective observational cohort study was conducted by sequentially selecting patients reviewed in 2020, with a diagnosis of confirmed PoTS in accordance with the Heart Rhythm Society Consensus Statement (7), at a single publicly funded neuro-otology tertiary clinic in London, United Kingdom. Institutional Ethics approval was obtained [IRAS ID: 257283; Research Ethics Committee (REC) reference number: 20/EM/0112], and the study protocol and execution were informed by discussion with volunteer patient representatives from PoTS UK.

### Subjects

Electronic clinical case notes were reviewed to identify patients with a diagnosis of PoTS, confirmed by a neuro-cardiology specialist. These patients were then compared, where appropriate, to an age- and sex-matched control group of new patients attending the Balance Clinic during the same period, who did not have confirmed or suspected PoTS.

### Outcome measures

Demographic data, the presence of defined vestibular symptoms (head motion-triggered dizziness, visually induced dizziness, spinning vertigo, positional vertigo, unsteadiness) (8), and examination findings were collated. Common PoTS-associated conditions were also recorded—potentially relevant to balance assessment and treatment. Specifically, migraine and vestibular migraine were diagnosed according to the IHS criteria (9) and the Barany Society criteria (10), respectively. Both definite and probable vestibular migraine were included. Other comorbidities (depression, anxiety, chronic pain, and hypermobility) were recorded as present if they were coded in the patient's health record.

Validated symptom questionnaires were recorded, specifically the Headache Impact Test (HIT6) (11), Generalized Anxiety Disorder Questionnaire (GAD-7) (12), Situational Vertigo Questionnaire (SVQ) (13), Dizziness Handicap Inventory (DHI) (14), and Disability Rating Scale (DRS) (15).

Examination findings, including Dix-Hallpike positional testing and clinical static and dynamic balance, were recorded. Specifically, static balance was recorded using the modified clinical test of sensory integration in balance (16). Dynamic balance was assessed by visual inspection of gait and tandem gait over 10 steps, with any side step or gap of more than 2 cm counted as an error, and with the best effort on three trials considered. Vestibular diagnostic test findings obtained from the six canal video head impulse test (VHIT), videonystagmography (VNG), oculomotor, and bithermal water stimulus VNG caloric assessments were recorded. Specifically, VNG was conducted according to standard protocol (17); gaze testing was performed in central and lateral gaze (+/– 30°) to assess for nystagmus with and without fixation. For smooth pursuit testing, participants were required to follow a target moving in a sinusoidal fashion at 0.2, 0.3, and 0.4 Hz. Horizontal saccadic testing employed a projected target moving randomly over a 5°-30° arc. Optokinetic testing subjected participants to remain stationary while the visual surround revolved around their vertical axis at a speed of 40°/s, alternating direction every 5–10 s for ~30 s. VHIT was considered abnormal if gain was below 0.8 for lateral canals and below 0.7 for vertical canals, combined with consistent grouped catch-up saccades. The caloric test was performed and recorded according to national guidelines (18).

Treatment recommendations for migraine management and subsequent referral for vestibular rehabilitation were noted in the medical record. National guidelines for migraine from the British Association for the Study of Headache were followed (19). Patients were asked to provide a Global Impression of Change on medication (overall benefit—neutral—overall worse), and a decision to stay on medication or not was documented. In terms of physiotherapy, benefit was additionally determined by the proportion benefitting from the intervention provided and determined by a change (decrease) on the Dizziness Handicap Inventory of more than 8 points before and after therapy.

All clinical assessments were performed by Consultant Audiovestibular Physicians or Highly Specialist Physiotherapists, and formal vestibular testing was conducted by specialist Audiologists, working collaboratively in a team within a large specialist tertiary Multidisciplinary Balance Clinic.

Statistical analysis was done using Statistical Package for the Social Sciences (SPSS) version 27.0 IBM Corp, including *t*-tests and chi-squared tests, with multiple hypothesis correction applied (Bonferroni).

## Results

A total of 80 eligible patients with PoTS were identified following the exclusion of non-consenting individuals. The cohort was predominantly female (74 of 80; 93%) with age ranging between 18 and 65 years and a mean age of 35.3 years (standard deviation, SD = 12.1). There were 52 control patients (48 women, 92%) with age ranging between 25 and 60 years and a mean age of 37 years (SD = 9.6). The groups showed no significant difference for sex (chi-squared test: 0.002; d*f* = 1; *p* = 0.9675) and age (*t*-test, *p* = 0.3207, *t* = 0.9968, d*f* = 129). The primary diagnosis in the control group was benign paroxysmal positional vertigo (*n* = 10), acute unilateral vestibular loss or its sequelae (*n* = 9), vestibular migraine (*n* = 10), persistent postural perceptual dizziness (*n* = 11), and other mixed diagnoses in the rest (*n* = 12).

The results related to symptoms, examination, comorbidity, and vestibular tests are listed in Table 1. Table 2 presents the questionnaires for the PoTS and control cohorts. Vestibular symptoms most commonly recorded in PoTS included unsteadiness (84%), and there were also relatively high rates of positional vertigo (68%) followed by spontaneous vertigo (48%). Comparison data for controls are given in Table 1. Controls had higher rates of head motion-triggered dizziness, with vertigo, whereas positional vertigo and unsteadiness were higher in the PoTS cohort.

**Table 1:** Prevalence of vestibular symptoms (not observed during postural induced tachycardia), comorbidities, and proportion of abnormality in examination and vestibular function testing in both controls and PoTS patients.

**Feature**	**PoTs group**		**Control group**		***P*-value**
	**Number assessed**	**Present/abnormal** ***n*** **(%)**	**Number assessed**	**Present/abnormal** ***n*** **(%)**	**Significance at 0.004 using Bonferroni correction**
Head Motion triggered dizziness	80	3 (4)	52	20 (40)	*p* < 0.0001^*^
					χ^2^ = 26.39
Visually induced dizziness	80	34 (43)	52	12 (24)	*p* = 0.0221
					χ^2^ = 5.24
Vertigo	80	38 (48)	52	19 (38)	P= 0.003^*^
					χ^2^ = 13.233
Unsteadiness	80	67 (84)	52	16 (32)	*p* = 0.0001^*^
					χ^2^ = 32.41
Positional vertigo	80	54 (68)	52	16 (32)	*p* < 0.0001^*^
					χ^2^ = 17.07
**Comorbidity**					
Migraine	80	67 (84)	52	23 (46)	*p* < 0.00005^*^
					χ^2^ = 18.12
Vestibular migraine	80	24 (30)	52	10 (20)	*p* = 0.3171
					χ^2^ =1.001
Chronic pain conditions	80	16 (20)	52	8 (16)	*p* = 0.54
					χ^2^ = 0.369
Depression	80	9 (11)	52	6 (12)	*p* = 0.9593
					χ^2 =^ 0.003
Anxiety	80	12 (15)	52	12 (24)	*p* = 0.2398
					χ^2^ =1.382
EDS/hypermobility	80	47 (59)	52	1 (2)	*p* = 0.0001^*^
					χ^2^= 43.98
**Examination**					
Dix-Hallpike positional test	65	9 (14)	52	5 (10)	NT
Static balance	64	14 (22)	52	7 (14)	*p* = 0.24
					χ^2^ =1.3698
Dynamic balance	61	13 (21)	52	5 (10)	*p* = 0.90
					χ^2^ =2.8675
**Vestibular function testing**					
VHIT	64	1 (2)	50	2 (4)	NT
VNG	64	1 (2)	47	3 (6)	NT
VNG caloric canal paresis	24	5 (19)	4	1 (25)	NT
VNG caloric directional preponderance	24	8 (22)	4	1 (25)	NT
CVEMP	2	2 (0)	0	0	NT

Degrees of freedom = 1 for all tests.

The asterisk symbol (*) indicates a *p* value of statistical significance at < 0.004.

SD, standard deviation; NT, not tested (see “Methods” section for further explanation); VHIT, video head impulse test; VNG, videonystagmography; CVEMP, cervical vestibular evoked myogenic potential.

**Table 2:** Questionnaire scores in PoTS and control groups.

**Validated instrument**	**PoTs group**		**Control group**		***P*-value**
	***n***	**Mean (SD)**	***n***	**Mean (SD)**	**Significance at 0.004 using Bonferroni correction**
Dizziness handicap inventory	51	54 (23)	26	45 (20)	*p* = 0.09
					*t* = 1.6453
					d*f* = 73
Situational Vertigo Questionnaire (SVQ)	43	1.8 (1.2)	15	1.00 (1.1)	*p* = 0.02
					*T* = 2.3133
					d*f* = 56
Headache impact HIT6	47	55 (18.3)	21	45 (23.9)	*p* = 0.07
					*T* = 1.8297
					d*f* = 66
Disability Rating Scale (DRS)	41	3.1 (1.6)	23	2.4 (1.1)	*p* = 0.0016^*^
					*T* = 3.3034
					d*f* = 62
Generalized anxiety disorder (GAD-7)	38	8.2 (12.2)	21	6.4 (5.8)	*p* = 0.525
					*T* = 0.6358
					d*f* = 57

*n*, number in each group; SD, standard deviation.

The asterisk symbol (*) indicates a *p* value of statistical significance at < 0.004.

The symptom questionnaires in the PoTS group demonstrated an SVQ mean score of 1.8 (SD = 1.2), Dizziness Handicap Inventory mean score of 54 (SD = 22.9), GAD7 mean score of 8.2 (SD = 12.2), HIT-6 mean score of 54.7 (SD = 18.3), and DRS mean score of 3.1 (SD = ±1.6) (Table 2). PoTS patients had higher scores than controls in all measures, which was statistically significant (*p* = 0.0016) for the Disability Rating Score. For the SVQ, the *p*-value was 0.02, but this was not considered statistically significant when multiple hypothesis correction was applied.

Due to restrictions placed by the COVID-19 pandemic during this data collection phase, face-to-face balance examination and vestibular function testing could not be performed for all patients. Of those assessed, 21% and 22% demonstrated abnormal dynamic or static balance, respectively, and 14% exhibited a positive Dix–Hallpike test. Comparison figures for the control group are provided in Table 1. However, statistical tests were not completed due to low absolute numbers of abnormal findings.

A total of 39 PoTS cases (49%) had migraine treatment and a care plan. In 8 of these 39 cases, this included using a beta blocker, which can be effective for both PoTS and migraine. Other medications offered included nortriptyline or amitriptyline (*n* = 11), topiramate (*n* = 3), candesartan (*n* = 2), and other specific advice in the rest (*n* = 15) which included use of other prescription medicines, management of menstrual migraine, guidance on acute attack management with medications, lifestyle modifications, and possible evidence-based nutritional supplements or other management strategies.

Of the 39 given migraine management advice, follow-up data (at least 12 weeks following intervention recommendation) were available for 18 patients; 9 patients reported a noticeable migraine improvement reporting both a favorable Global Impression of Change and continued their medication [including nortriptyline, candesartan, beta blockers, topiramate, frovatriptan, and gabapentin (NB: gabapentin was previously included in national guidelines for migraine management, though it has since been removed)]. The remaining nine patients required further intervention. A total of 46 PoTS patients (58%) were offered formal vestibular physiotherapy. Of the 30 patients with available follow-up data (at least 12 weeks following intervention recommendation), 19 patients (63%) demonstrated clinically significant benefit according to the definition, and the remaining 11 patients (37%) reported no benefit.

For comparison, in the control group, 46/50 (92%) underwent vestibular rehabilitation and 14/50 (28%) received additional migraine advice.

## Discussion

Our study reports a real-world neurotological perspective in a large cohort of PoTS patients who have also received expert neuro-cardiac evaluation.

Compared to the general population, where 6.5% of men and 18.2% of women experience migraines, the PoTS population in previous studies demonstrates a greater prevalence of migraine of up to 40% (5, 20, 21). It is noted that studies investigating the prevalence of headaches in PoTS seldom identify each headache subtype to determine a representative migraine prevalence for comparison (4, 10, 12). Similarly, dizziness is rarely characterized in detail from a neuro-otological perspective in studies on PoTS.

A relatively high proportion of characteristically “vestibular” symptoms, including visually triggered dizziness, spontaneous vertigo, and positional vertigo, were reported in our cohort.

We also report a migraine prevalence of 84%, which is evidently greater than previously described. Although this disparity is partly attributed to cohort size and referral bias, since our clinic is known for expertise in managing migraine-related dizziness conditions, there was a significantly greater prevalence of migraine in our PoTs population compared to our comparator group.

Although our primary aim in this study was to describe Neuro-otological findings in a PoTS cohort, for a reference point, we compared our PoTS cohort to an age- and sex-matched control group of other dizzy patients reviewed in our clinic. It should be remembered that this control population is atypical for the clinic population due to the need for age and sex matching, which, in this case, necessitates a higher proportion of younger adult women. Compared to the controls, the PoTS cohort had a higher prevalence of vertigo, unsteadiness, and positional vertigo, while the control group had a higher prevalence of head motion-triggered dizziness. This higher prevalence is likely due to a control group with a greater number of younger women. The exclusion of older adults may have reduced the number of individuals with BPPV, who may experience more vertigo.

The diagnosis of Ehlers–Danlos syndrome is complex, and we did not distinguish these in the analysis. Hypermobility was confirmed in the PoTS clinic using the Beighton score, with a cutoff of 4. Visually triggered dizziness was similar in both groups. The rate of visually triggered dizziness in the PoTS group is noteworthy, with 43% describing visually triggered dizziness, similar to the control group. Notably, none of these symptoms are directly attributable to the cardiovascular manifestations of PoTS, suggesting the possibility that there are other mechanisms contributing to dizziness in this group. Since migraine is a recognized comorbidity in PoTs, it is likely that migraine (or vestibular migraine) is responsible for some of these symptoms.

Clinicians diagnosed VM according to recognized criteria in 30% of our PoTs patients, a higher rate than the comparison group (20%), but not statistically significantly different.

Like PoTS, vestibular migraine manifests symptomatically with both migraine and dizziness, demonstrates a strong female preponderance, and is underdiagnosed by healthcare professionals (22). When considering diagnostic criteria for VM (10), it is necessary for clinicians to exclude other causes of symptoms, and this may be challenging in the presence of a comorbidity that is also associated with both dizziness and migraine, such as PoTS. We consider if this may be a reason why the prevalence of migraine was very high (84%) in our cohort, but only 30% were diagnosed with vestibular migraine. Further research to develop instruments to assess dizziness and migraines, particularly vestibular migraine, in complex populations such as those with PoTS would be beneficial.

Approximately 59% of our PoTS cohort have a diagnosis of hypermobility, similar to the combined finding of 55% described by Miller et al. in the first cross-sectional prospective study evaluating the prevalence of hypermobile EDS and general joint hypermobility in PoTS (23). To the best of our knowledge, despite being well documented as an association, it is not known why PoTS and EDS/hypermobility syndrome overlap, nor why there is an association with migraine. Furthermore, we identified the prevalence of chronic pain (20%), anxiety (15%), and depression (11%), all of which may influence illness perception; particularly, anxiety may potentially cause or complicate dizziness.

Our PoTS cohort also had an abnormal Dix-Hallpike positioning test in 14%, including cases of benign paroxysmal positional vertigo, again confirming that the mechanism of dizziness in this cohort can be complex and multifaceted. The positive Dix–Hallpike test finding may have several explanations. Benign paroxysmal positional vertigo and migraine can both cause vertigo and nystagmus on positional tests. Since approximately a third of the PoTS population experience recurrent syncopal episodes (24), a predisposition to head trauma and thereby an association with benign paroxysmal positional vertigo may be theorized. This is a presentation the authors have observed; however, the proposed mechanism remains speculative. A similar rationale may apply to the abnormalities observed in caloric and VHIT testing, which could, in part, be due to a post-traumatic etiology.

Similarly, clinical static and dynamic balance tests were frequently abnormal. This may be secondary to the influence of other comorbidities, such as joint hypermobility, or directly related to the pathophysiology of PoTS, such as in cases associated with a lower limb small fiber neuropathy (25, 26). Further research is warranted to elucidate the mechanisms of impaired balance control in this population.

A considerable proportion were offered migraine management advice and continued the medication at follow-up. This is consistent with our clinical experience, where migraine management, for which several efficacious evidence-based treatments exist, may be easily neglected in the context of a patient population with significant comorbidity. Addressing migraine has the potential to improve quality of life. Similarly, vestibular physiotherapy is beneficial for managing symptoms in selected cases. This observation demonstrates the value of a multidisciplinary approach to assessing and managing dizziness in individuals with PoTS. While it may not be practical for all patients to access specialist neuro-otological or neurological assessments, research could be directed toward developing standardized assessment tools to identify which patients may benefit from specific interventions.

The primary limitations of this study are its retrospective design, observational nature in relation to clinical practice, and referral bias, the latter of which is typical of a specialist clinic cohort and may limit generalizing findings to the PoTS population more widely. Although our study was conducted during the COVID-19 pandemic, which has influenced data completeness, our findings still illustrate the heterogeneity of dizziness in PoTS; several atypical dizziness conditions, often considered atypical of PoTS, were additionally identified within this study. We were able to carry out clinical neuro-otological assessments in a cohort that had been well characterized from a neuro-cardiological perspective, providing a grounding for future work in this area.

## Conclusion

Our findings reflect the challenges of distinguishing dizziness phenotypes, including vestibular disorders and vestibular migraine, within the PoTS population. Vestibular migraine appears to be common in patients with PoTS. We advocate judicious clinical assessment of dizziness in PoTS to identify non-orthostatic mechanisms that may cross traditional disciplines and necessitate contributions from neurologists, vestibular specialists, and cardiologists, among others. We provide a basis for further prospective research to elucidate the multifaceted nature of PoTS-associated dizziness and for the development of clinical tools to assess vestibular migraine and migraine in complex patient populations.

## Data Availability

The raw data supporting the conclusions of this article will be made available upon reasonable request and subject to institutional approvals. Requests to access the datasets should be directed to louisa.murdin@gstt.nhs.uk.
